# Efficacy of Acupuncture and Moxibustion in Alopecia: A Narrative Review

**DOI:** 10.3389/fmed.2022.868079

**Published:** 2022-06-09

**Authors:** Andraia R. Li, Laura Andrews, Alexis Hilts, Manuel Valdebran

**Affiliations:** ^1^Department of Dermatology and Dermatologic Surgery, The Medical University of South Carolina, Charleston, SC, United States; ^2^Kirk Kerkorian School of Medicine at UNLV, Las Vegas, NV, United States; ^3^Department of Pediatrics, The Medical University of South Carolina, Charleston, SC, United States

**Keywords:** acupuncture-therapy, alopecia-etiology, alternative medicine (CAM), hair loss (alopecia), moxibustion acupuncture, acupoint, systematic review

## Abstract

Acupuncture is the practice of applying needles to target specific pressures points in the body. Since originating in China, acupuncture has been practiced for thousands of years to treat numerous conditions including chronic pain and mood disorders. Alopecia is a common dermatologic condition associated with psychological distress and decreased quality of life. Although it remains underexplored in western medicine, recent evidence suggests that acupuncture may be efficacious in the treatment of alopecia. In this review, we discuss the available evidence describing the efficacy of acupuncture or moxibustion alone (ACU) and in combination with other traditional and alternative interventions (ACU + TRAD) for hair loss. Additionally, the proposed physiologic mechanisms, targeted acupuncture points, and the benefits and barriers to treatment will be further described. An exploratory search using PubMed, EMBASE and Scopus databases was performed for studies that evaluated the effect of acupuncture and moxibustion on alopecia. In these studies, both ACU and ACU + TRAD were efficacious for numerous etiologies of hair loss including alopecia areata, androgenetic alopecia, and seborrheic alopecia. Given their ability to modulate the immune system, as well as neuronal networks associated with emotional cognition, the most frequently targeted acupoints were ST 36, GV 20, and LR 3. The proposed mechanistic effect is dependent upon disease etiology and is theorized to be twofold: reduction of inflammation and decrease in testosterone levels. The limited side effect profile of acupuncture makes it an advantageous treatment option, however, factors including cost, time, limited access, and aversion to needles may serve as barriers to treatment.

## Introduction

Acupuncture and moxibustion have been commonly practiced in traditional Chinese and Eastern medicine over the past three thousand years (1). Acupuncture is the practice of applying needles to target specific pressure points while moxibustion thermally stimulates these meridian points by burning dried mugwort herbs (2). Health benefits associated with acupuncture include alleviation of post-operative and chemotherapy-associated nausea and vomiting and post-operative dental pain (3). Acupuncture was not widely practiced in the United States until the 1970s and the acupuncture needle was not recognized as a medical device by the Food and Drug Administration (FDA) until 1996 (4, 5). Since then, acupuncture practice and research has rapidly advanced with recent evidence suggesting that acupuncture may also be efficacious in the treatment of alopecia (6).

Alopecia is a common dermatologic condition associated with psychological distress and decreased quality of life (7). Treatment of alopecia is dependent on etiology. Androgenetic alopecia is estimated to affect 50% of men by age 50, and 38% of women older than 70 years (8). Minoxidil and finasteride are currently the only FDA-approved treatments for androgenetic alopecia. Although both treatments have been shown to be effective in promoting hair growth in men with androgenetic alopecia, the efficacy of these treatments may be inflated considering publication bias (9). Potential side effects of low-dose minoxidil and finasteride include hypertrichosis and gynecomastia which may be prohibitive to their use (10, 11). Alopecia areata is a type of inflammatory and non-scarring hair loss that is estimated to affect 2% of the population worldwide with increasing prevalence over time (12). A variety of topical, systemic and injectable agents are used in the treatment of alopecia areata including corticosteroids and several immunosuppressive agents. Despite the array of treatment options, these methods produce variable outcomes, and no current treatment both induces and sustains remission in alopecia areata (12, 13).

The treatment of alopecia continues to prove challenging as results are inconsistent and efficacy is often limited by side effect profile. Given these challenges, the relative safety of acupuncture and the heterogeneity of previously published reports, we reviewed the evidence to assess the efficacy of acupuncture and moxibustion in the treatment of alopecia. We aimed to assess the most commonly targeted acupoints and the current understanding of the potential mechanism of action in order identify future directions for acupuncture in the treatment of hair loss.

## Methods

An exploratory search using PubMed, EMBASE, and Scopus databases was performed to identify studies that evaluated the effect of acupuncture and moxibustion on hair loss. The search strategy was comprised of a combination of medical subject headings and keywords for acupuncture (e.g., acupressure, acupoint, electroacupuncture, laser acupuncture, moxibustion, and meridians) and hair loss (e.g., alopecia areata, seborrheic alopecia, androgenic alopecia, male pattern baldness, folliculitis decalvans, lichen planopilaris, telogen effluvium, traction alopecia, frontal fibrosing alopecia, and central centrifugal cicatricial alopecia). Case-reports, observational studies, and clinical trials assessing the efficacy of acupuncture alone (ACU) or in combination with other traditional therapies (ACU + TRAD) for the treatment hair loss were eligible for inclusion. Non-English studies and articles that did not report therapeutic outcomes in response to acupuncture were excluded.

## Review of the Current Evidence

The main findings of the 8 studies in acupuncture and hair loss are summarized in Table 1. These etiologies included alopecia areata, androgenetic alopecia, seborrheic dermatitis and perifolliculitis capitis abscedens et suffodiens (PCAS). Duration of needle retention ranged from 2 to 30 min while the number of total treatment sessions ranged from 3 to 50. Acupuncture was performed at least three times a week in 5 studies while the interval between treatments ranged from 1 to 4 weeks in the remaining 3 studies. The most commonly targeted acupoints were ST 36 (*n* = 4) (14–17), GV 20 (*n* = 3) (15, 17, 18), and LR 3 (*n* = 3) (15–17). Details of the study protocols and targets acupoints are summarized in Table 2.

**TABLE 1:** Efficacy of acupuncture and moxibustion for alopecia.

Study	Study design, number of subjects	Interventions	Etiology of alopecia	Mean age (SD), years	Assessed outcomes	Definition of assessed outcomes	Duration of treatment	Main findings	Adverse events	Key takeaways
Gao et al. (14)	RCT, 84	Catgut embedment-Moxibustion-Bloodletting with plum blossom needling (Cohort 1) vs. Finasteride 1 mg/day qd for 3 months with 5-day rest between each course of treatment (Cohort 2)	Androgenetic	NA	Testosterone, Estradiol, *D*-value HAMD reductive rate composited as the total effective rate (TER)	*D*-value HAMD reductive rate equal to (value before treatment - value after treatment)/value before treatment × 100 where value is the ratio of testosterone/estradiol	3 months	In the catgut embedment-Moxibustion-Bloodletting with plum blossom needling cohort, testosterone and estradiol decreased from 1259.5 ± 1009.5 ng/dL and 62.09 ± 50.45 pg/mL to 405.1 ± 483.39 ng/dL and 33.38 ± 29.32 pg/mL, respectively. Testosterone and estradiol decreased in the Finasteride cohort as well (1247 ± 554 ng/dL and 58.74 ± 54 pg/mL to 555.19 ± 944.26 ng/dL and 50.21 ± 35.9 pg/mL) but to a lesser extent. Testosterone and estradiol were significantly lower in the ACU + TRAD group (*p* < 0.05). Based on reduction in HAMD scores, TER in the ACU + TRAD cohort was significantly greater than the finasteride cohort (97.62 vs. 83.3%. *p* < 0.05).	NA	The catgut embedment-Moxibustion-Bloodletting with plum blossom needling resulted in significant reduction in testosterone and estradiol compared to finasteride. Clinical images showed improvement in alopecia areata but investigators did not quantify differences in hair loss and growth between groups. The efficacy of acupuncture alone cannot be assessed in this study as investigators used a combination of traditional treatments.
Jin et al. (18)	RCT, 70	Combined electroacupuncture and acupoint injection of Mecobalamin (Cohort 1) vs. seven-star needle tapping once every day and a couple of times of rubbing the affected area using a fresh ginger piece daily for 30 days (control) (Cohort 2)	Alopecia areata	35 (4) in Cohort 1, 39 (5) in Cohort 2	Recovery, Marked effect, Failure, composited as the TER.	Recovery was defined as complete growth of new normal hair and a negative test of hair pull test. Marked effect was defined as >50% anagen hair growth, thickening of the new anagen hairs and negative result of hair pulling test. Failure was defined as no new anagen hairs; new hair growth was less than 10% or concurrent hair growth and hair loss.	30 days	In the combined electroacupuncture and acupoint injection of Mecobalamin cohort, recovery, marked effect and failure was observed in 15, 18, and 2 patients, respectively, for a composite TER of 94.3%. In the seven-star needle tapping (control) cohort, recovery, marked effect and failure was observed in 10, 17 and 8 patients. The TER in this cohort was significantly less than the electroacupuncture group (94.3 vs. 77.1%, *p* < 0.05).	NA	Electroacupuncture with acupoint injection was more effective in eliciting hair growth than the seven-star needling technique (control) group. Use of electroacupuncture at specific acupoints in combination with acupoint injection may be more efficacious than application of seven-star needling at areas of hair loss in the treatment of alopecia areata. The efficacy of acupuncture alone cannot be assessed in this study as investigators used a combination of traditional treatments.
Kawashima et al. (20)	CR, 1	Japanese Kampo medicine (JKM) formulas in combination with acupuncture. After “relapse,” (163 days after discontinuing treatment) self-administration of pine-needle acupuncture was initiated in combination with the JKM formulas.	Alopecia areata	34	Severity of Alopecia Tool (SALT) score	SALT score measures the percentage of hair loss in 4 areas: vertex (40%), right and left profile (18% each) and posterior scalp (24%) to provide a composite score. A decrease in the SALT score indicates regrowth.	327 days	SALT score was 19% at diagnosis and decreased to 9% on day 124 of treatment with JKM and acupuncture. The patient experienced complete resolution of hair loss on day 159 and treatment was discontinued. Symptoms returned on day 322 (SALT 13%) and the patient resumed JKM in combination with self-administered pine-needle acupuncture. Sequential SALT scores were 11%, 5% (>50% recovery from baseline at relapse), 4%, and 2% (>75% recovery) on days 392, 420, 448, and 490, respectively. The patient remained in remission and is receiving JKM formulas as of 4/2021.	NA	JKM and acupuncture was associated with reduction in hair loss in a patient with alopecia areata. JKM in combination with self-administered pine-needle acupuncture was also efficacious in this case and may be a more sustainable treatment regimen for maintaining long-term results.
Li et al. (22)	RCT, 87	Plum blossom needling with qi-invigorating botanical therapies (Cohort 1) vs. Oral cystine 50 mg TID, Vitamin B6 10 mg TID (Cohort 2)	Seborrheic dermatitis	NA	Testosterone, Estradiol, Hair loss, Hair growth	Quantitative scoring of hair loss: Minimal hair loss (<100 hairs) was scored as 0 points; loss of 150–100 hairs was considered a small amount of hair loss and was scored as 2 points; loss of 200–150 hairs was considered moderate and scored as 4 points; and loss of ≥200 hairs was considered severe and scored as 6 points. Quantitative scoring of hair growth (higher score indicates less regrowth): Complete regrowth in area of hair loss, with normal hair thickness and color after 2 months of treatment was scored 0 points; >2/3 new hair regrowth in areas of hair loss but with non-uniform hair color and thickness was scored 2 points; 1/3 hair regrowth in areas of hair loss with pale hair color and soft texture was scored 4 points; no regrowth or presence of few hairs was scored 6 points. Evaluations were performed after the patients washed their hair in the morning,	2 months	Patients in the plum blossom needling with traditional Chinese medicine experienced a greater reduction in testosterone than the medication cohort (6.38 ± 1.12 vs. 8.92 ± 1.62 μg/L, *p* < 0.05). Additionally, estradiol in the ACU + TRAD was significantly greater than the medication cohort after treatment (72.02 ± 4.13 vs. 68.63 ± 5.29 pg/mL, *p* < 0.05). There was no significant difference in testosterone and estradiol between cohorts at baseline. Likewise, both cohorts experienced comparable degrees of hair loss and growth at baseline. Following treatment, the patients in the ACU + TRAD cohort had a significantly lower hair loss score than the medication group (1.69 ± 0.96 vs. 2.65 ± 1.06), indicating less hair loss. Quantitative hair growth score was significantly lower in the ACU + TRAD cohort than the medication cohort (2.21 ± 1.16 vs. 3.56 ± 1.2), indicating less regrowth in the medication group.	The incidence of emotional disorders (11.6 vs. 0%) and sexual dysfunction (9.3 vs. 0%) was significantly lower in the ACU + TRAD group (*p* < 0.05).	Plum blossom needling in combination with traditional Chinese medicine was associated with a greater reduction in testosterone and increase in estradiol than cystine and vitamin B6. Additionally, the ACU + TRAD treatment resulted in less hair loss and promoted more growth in patients with hair loss secondary to seborrheic dermatitis. However, patients in the ACU + TRAD also experienced higher rates of sexual dysfunction and emotional disturbances than the medication group during treatment. It is unknown how the efficacy of acupuncture for hair loss in seborrheic dermatitis compares to first-line agents including topical antifungals and corticosteroids.
Su et al. (23)	CR, 3	5% 5-aminolevulinic acid-based photodynamic therapy (ALA-PDT) combined with oral isotretinoin (10 mg) TID. Fire needle intervention was used as a pretreatment for ALA-PDT.	Perifolliculitis capitis abscedens et suffodiens (PCAS)	27.7 (13.3)	Qualitative description	NA	2 weeks to 4 months	At 2 weeks, 2 of 3 cases reported a significant reduction in nodules, cysts and pain after initiation of treatment. Resolution of PCAS and hair regrowth was observed at 6, 8 weeks and 4 months in the 3 cases. No patients had recurrence with minimum one year follow up. Two of 3 cases experienced no recurrence at 2 year follow-up as well.	Local redness, swelling and pain after PDT, which resolved in a week. All 3 patients experienced dry lips, mucous membranes and skin but reported symptoms did not affect their quality of life.	Isotretinoin is first line for moderate-severe refractory PCAS but works slowly and is known to have harmful side effects with prolonged use. In these 3 cases, combining isotretinoin with PDT pretreated by fire needle improved symptoms early, shortened the course of treatment, consolidated efficacy and reduced disease recurrence.
Wu et al. (15)	CR, 1	Acupuncture	Alopecia areata universalis	62	Qualitative description	NA	3 months	After the 13th session, there was an increase in hair on the scalp with small patches of black vellus hair present. After 3 months of treatment, regrowth occurred in all areas of hair loss. Results were maintained at 2 month follow-up.	NA	Acupuncture was associated with complete regrowth in areas of hair loss after 3 months of treatment in a patient with alopecia universalis.
Zhang et al. (16)	CR, 1	Medicated thread moxibustion using the traditional Zhuang medicine method	Alopecia areata	36	Qualitative description	NA	3 months	One week after treatment, a small amount of vellus hair regrew in areas of hair loss. At 3 weeks, patches of hair loss were covered with hair of different lengths and colors. 4 weeks after treatment (final session), complete regrowth was observed in area of hair loss albeit with decreased density compared to the circumferential area. Results were maintained at 3 months follow-up with negative hair pull test.	NA	Moxibustion was associated with complete hair regrowth at 4 weeks in a patient with alopecia areata.
Zhu and Wu (17)	RCT, 78	Acupuncture and plum-blossom needle therapy (Cohort 1) vs. Cystine tablets 0.1 g TID, Vitamin B1 20 mg TID and 2% Minoxidil Solution applied topically BID (Cohort 2)	Alopecia areata	NA, age ranged from 17–60 years	Cure, Remarkable effect, Effect, Failure, composited as the TER.	Cure was defined as 100% new hair growth, with dense distribution, normal color, and a negative hair-pulling test. Remarkable effect was defined as 70% of new hair growth, with normal density, size and color. Effect was defined as 30% of new hair growth, including fine hair and gray hair, and cessation of additional hair loss with treatment. Failure was defined as less than 30% of new hair growth or continual hair loss after 4 courses of treatment.	4 months	In the acupuncture and plum-blossom needling cohort, the cure rate and TER was 58.1 and 97.7%, respectively. This was significantly greater than the corresponding cure rate and TER in the medication cohort (34.3 and 77.1%, respectively. In the ACU + TRAD group, cure, remarkable effect, effect and failure was reported in 25, 13, 4, and 1 patient compared to 12, 11, 4, and 8 patients in the medication group.	NA	Acupuncture in combination with plum blossom needling may be more efficacious than cystine, vitamin B1 and 2% topical Minoxidil solution in patients with alopecia areata. The majority of patients in the acupuncture cohort experienced complete hair regrowth vs. only one-third in the medication group after 4 months of treatment.

*Abbreviations: Pro, prospective; RCT, randomized controlled trial; CR, case report; BID, twice a day; TID, three times a day; QD, once a day; SD, standard deviation; NA, not available; TER, total effective rate; JKM, Japanese Kampo medicine; SALT, severity of alopecia tool; PCAS, perifolliculitis capitis abscedens et suffodiens; ALA-PDT, 5-aminolevulinic acid-based photodynamic therapy; ACU, acupuncture alone; ACU + TRAD, acupuncture in combination with traditional therapy.*

**TABLE 2:** Treatment specifications and details of eligible studies.

Study	Country	Intervention	Number of acupuncture sessions	Duration of sessions (min)	Acupuncture points targeted	Interval between acupuncture treatments	Additional treatment details
Gao et al. (14)	China	Catgut embedment-Moxibustion-Bloodletting with plum blossom needling (Cohort 1) vs. Finasteride 1 mg/day qd for 3 months with 5-day rest between each course of treatment (Cohort 2)	Catgut embedment: 3, Moxibustion: 50 (10 courses, with each course consisting of 5 sessions), Bloodletting with plum blossom needling: 4	Moxibustion: 30	Acupoints used for catgut embedment: Dong’s acupuncture point (defined as 1.5 cun or 1.5 times the distance between DIP and PIP of the middle finger below SP 9), Minghuang (T 88.12) and Zusanli (ST 36). Acupoints used for moxibustion: Qihai (CV 6), Guanyuan (CV 4)	Catgut embedment: performed once a month, for a total of 3 sessions. Moxibustion: performed every other day for a total of 50 sessions. Bloodletting with plum blossom needling: performed. every other week	During catgut embedment, catguts were sterilized and embedded onto acupoints using disposable needles. Moxibustion was performed using moxa sticks (Yaowang Pharmacy Limited Co., Anguo, China) applied into three-hole and four-hole moxa boxes placed on acupoints of interest. For bloodletting, plum-blossom needling was used in areas of hair loss.
Jin et al. (18)	China	Combined electroacupuncture and acupoint injection of Mecobalamin (Cohort 1) vs. seven-star needle tapping once every day and a couple of times of rubbing the affected area using a fresh ginger piece daily for 30 days (control) (Cohort 2)	Approximately 26 (sessions performed 6 out of 7 days a week, observed over 30-day period)	30	None, scalp pierced in area of hair loss. Treatment for some patients was augmented by puncturing Baihui (GV 20), Fengchi (GB 20) and Taiyang (EX-HN 5). Other acupoints were targeted depending on patient specific chief-complaints (e.g., for anemia, use Zusanli (ST 36) and Xuehai (SP 10); for “qi stagnation” and “blood stasis,” use Taichong (LR 3), Xuehai (SP 10), Neiguan (PC 6) and Touwei (ST 8); for dizziness, use Shangxing (GV 23) and Zusanli (ST 36); for insomnia, use Shenmen (HT 7) and Sanyinjiao (SP 6); for lower back pain and tinnitus, use Shenshu (BL 23) and Taixi (KI 3)	Electroacupuncture cohort: Daily with 1-day interval after 6 consecutive days of treatment, seven-star needle tapping (control) cohort: daily self-administration	Acupuncture needles are first inserted into the muscular layer of the scalp, at the border of the affected hair loss area. Electroacupuncture is performed at a frequency of “200 times per minute.” Additionally, the needle is manipulated in a twirling manner for 1–2 min, for a minimum of 1 min, before withdrawing the needle to the superficial layer of the scalp where it is then retained for the remainder of the 30-min session. These steps are repeated on the opposite side of hair loss. Needles are kept apart to avoid short circuiting.
Kawashima et al. (20)	Japan	Japanese Kampo medicine (JKM) formulas in combination with acupuncture. After “relapse,” (163 days after discontinuing treatment) self-administration of pine-needle acupuncture was initiated in combination with the JKM formulas.	Not reported, 159 days with JKM + acupuncture, 238 days with JKM + self-administration of pine-needle	15 min for acupuncture, 2–3 min for pine-needle self-acupuncture	None, targeted area of hair loss (needles placed laterally from the edge of the lesion toward the center)	Performed weekly for acupuncture, once or twice weekly for pine-needle acupuncture self-administration	JKM consisted of pharmaceutical grade saikokaryukotsubor- eito extract (7.5 g/day; Kotaro Pharmaceutical Co., Ltd., Osaka, Japan) and rokumiga (7.5 g/day; Tsumura and Co., Tokyo, Japan). Disposable Seven-star Needles (Suzhou Acupuncture Goods Co., Ltd., Suzhou, China) were used for plum blossom acupuncture, which consistent of local stimulation with needles for 2–3 min once or twice each week.
Li et al. (22)	China	Plum blossom needling with “qi-invigorating superficies-consolidating” therapy (Cohort 1) vs. Oral cystine 50 mg TID, Vitamin B6 10 mg TID (Cohort 2)	10	10	None, scalp pierced in hair loss area until skin began to bleed lightly with needles coated with hair growth preparation consisting of cacumen biotae, *Rhizoma drynariae*, *Salvia miltiorrhiza*, white mulberry bark, and *Zanthoxylum bungeanum*	Performed every other day	“Qi-invigorating superficies-consolidating” therapy included the following: astragalus (30 g), saponin (10 g), forsythia (10 g), poria (10 g), aquilaria (10 g), stone acorus (5 g), acacia peel (30 g), *Platycodon grandiflorum* (10 g), *Chuanxiong rhizome* (10 g), Lignum millettiae (30 g), cork (5 g), *Tribulus terrestris* (15 g), Bauhinia peel (15 g), charred hawthorn (15 g), mulberry (15 g), *Polygonum multiflorum* (30 g), *Radix sileri*s (15 g), and fried atractylodes (10 g). Patients took 1 dose per day for a total of 14 doses.
Su et al. (23)	China	5% 5-aminolevulinic acid-based photodynamic therapy (ALA-PDT) combined with oral isotretinoin (10 mg) TID. Fire needle intervention was used as a pretreatment for ALA-PDT.	4	NA	None, targeted follicular papules, nodules, cysts and alopecia areas	Performed every 2 weeks	Acupuncture needles were sanitized under fire and used to pierce targeted areas including follicular papules, nodules, cysts and areas of hair loss. Purulent secretions were cleaned with. cotton swabs. Areas were then prepped with 5% 5-AMA and then incubated under light for 2 h at a wavelength of 630 ± 5 nm and an energy density of 70 mw/cm for 20 min. This was performed every 2 weeks for a total of 4 sessions. Isotretinoin was administered three times a day for 4 months.
Wu et al. (15)	China	Acupuncture	36	30	Baihui (GV 20), Taiyang (EX-HN 5), Fengmen (BL 12), Xinshu (BL 15), Ganshu (BL 18), Shenshu (BL 23), Zusanli (ST 36), Sanyinjiao (SP 6), Xuanzhong (GB 39), Taix (K 13), Kunlun (BL 60), Taichong (LR 3), Neiguan (PC 6), Waiguan (TE 5), Shenmen (HT 7), Hegu (L1 4), Tianshu (ST 25), Zhongwan (CV 12), Qihai (CV 6), Guanyuan (CV 4), Danzhong (CV 17), Juliao (ST 3), Shuigou (GV 26) and Chengjiang (CV 24)	Performed 3 times a week	
Zhang et al. (16)	China	Medicated thread moxibustion using the traditional Zhuang medicine method	14	NA, each acupoint was cauterized 2 times, once every 2 days for 4 weeks.	Kuihua (Points specific to Zhuang medicine method, location of the point dependent on the shape and size of local skin lesions on the body surface wherein a group of acupoints were selected along the periphery and midpoint of the body: Zusanli (ST 36), Xuehai (SP 10), Baihui (DU 20), and Taichong (LR 3)	Performed every other day	Held between the thumb and index finger, the exposed end of a medicated thread was lit under an alcohol lamp and applied to points of interest.
Zhu and Wu (17)	China	Acupuncture and pricking of plum-blossom needle therapy (Cohort 1) vs. Cystine tablets 0.1 g TID, Vitamin B1 20 mg TID and 2% Minoxidil Solution applied topically BID (Cohort 2)	40 (4 courses comprised of 10 sessions)	30	Major acupoints: Shenshu (BL 23), Ganshu (BL 18), Taixi (KI 3), Sanyinjiao (SP 6), Xuehai (SP 10), Geshu (BL 17), Zusanli (ST 36), Fengchi (GB 20), Baihui (GV 20), Shangxing (GV 23) and Shuaigu (GB 8). Adjunct acupoints: Neiting (ST 44) for alopecia above the forehead; Taichong (LR 3) for alopecia affected the vertex; Waiguan (TE 5) for alopecia affecting bilateral sides of the head; and Houxi (SI 3) for alopecia affecting the occiput.	Performed every other day	Needles were manipulated every 10 min using a twisting technique consisting of moving the needle back and forth between the thumb and the index finger. Plum-blossom needling in areas of hair loss was performed after acupuncture. The strength of the needling was dependent on the severity and the duration of hair loss.

*ALA-PDT, 5-aminolevulinic acid-based photodynamic therapy; JKM, Japanese Kampo medicine; BID, twice a day; TID, three times a day; NA, not available.*

### Acupuncture or Moxibustion Alone

In a case report by Wu et al., acupuncture was associated with complete regrowth in areas of hair loss 3 months after treatment in a patient with alopecia areata universalis with results maintained at 2 months follow-up (15). In a randomized controlled trial (RCT) of 78 patients with alopecia areata, Zhu et al. found traditional acupuncture in combination with plum blossom needle acupuncture was associated with higher cure rates of alopecia areata than oral cystine with vitamin B6 and 2% topical minoxidil (58.1 vs. 77.1%, *p* < 0.05) after 4 months of treatment (17). Zhang et al. also reported of a case of alopecia areata where medicated-thread moxibustion using the traditional Zhuang medicine method was associated with hair regrowth appreciable after 1 week of treatment with complete regrowth at 4 weeks and results maintained at 3 months (16).

### Acupuncture or Moxibustion in Combination With Other Therapies

#### Moxibustion With Catgut Embedment and Bloodletting Using Plum Blossom Needling

In a RCT by Gao et al., patients with androgenetic alopecia treated with moxibustion in combination with catgut embedment and bloodletting using plum blossom needling had significantly lower levels of testosterone (405.1 ± 483.39 vs. 555.19 ± 944.26 ng/dL, *p* < 0.05) and estradiol (33.38 ± 29.32 vs. 50.21 ± 35.9 pg/mL, *p* < 0.05) than patients in the finasteride cohort following treatment (14). Clinical images also showed improvement in alopecia areata in response to ACU + TRAD improvement, however, investigators failed to quantify differences in hair loss and growth between groups (14).

#### Electroacupuncture With Acupoint Injection of Mecobalamin

In a RCT of 70 patients with alopecia areata, Jin et al. found electroacupuncture in combination with acupoint injections of mecobalamin, a co-factor of vitamin B12 commonly used to treat peripheral neuropathy, was associated with a significantly greater total effective rate (TER) (94.3 vs. 77.1%, *p* < 0.05) than seven-star needle tapping in areas of hair loss after 30 days of treatment (18, 19).

#### Acupuncture With Japanese Kampo Medicine

Kawashima et al. report of a case of alopecia areata successfully treated with acupuncture in combination with Japanese Kampo medicine (JKM), a form of traditional Japanese herbal medicine (20, 21). Treatment resulted in reduction of Severity of Alopecia Tool (SALT) score from 19% at baseline to complete resolution of hair loss was observed on day 159 and treatment was discontinued. Symptoms returned on day 322 (SALT 13%) and the patient was directed to resume JKM in combination with self-administered pine-needle acupuncture in place of traditional acupuncture (20). On this regimen, the patient achieved >50% and >75% recovery from baseline at relapse on day 420 and day 490, respectively (20).

#### Plum Blossom Acupuncture With Qi-Invigorating Botanical Therapies

In a RCT of 87 patients with hair loss secondary to seborrheic dermatitis, Li et al. found plum blossom needling in combination with traditional Chinese medicine was associated with significantly less hair loss (1.69 ± 0.96 vs. 2.65 ± 1.06, *p* < 0.05) and more hair growth (2.21 ± 1.16 vs. 3.56 ± 1.2, *p* < 0.05) than oral cystine/vitamin B6 (22). Those in the ACU + TRAD group exhibited significantly lower levels of testosterone (6.38 ± 1.12 vs. 8.92 ± 1.62 μg/L, *p* < 0.05) and higher levels of estradiol (72.02 ± 4.13 vs. 68.63 ± 5.29 pg/mL, *p* < 0.05) compared to the medication group (22). Patients in the medication group reported higher rates of sexual dysfunction, which the authors defined as decreased sexual desire and erectile dysfunction (9.3 vs. 0%, *p* < 0.05), and emotional disturbances (11.6 vs. 0%, *p* < 0.05), than patients in the ACU + TRAD group during treatment (22).

#### Acupuncture With 5% 5-Aminolevulinic Acid-Based Photodynamic Therapy and Isotretinoin

Su et al. report of three cases of PCAS responding to acupuncture in combination with 5-aminolevulinic acid-based photodynamic therapy (ALA-PDT) and isotretinoin (23). Two of 3 cases reported a significant reduction nodules, cysts and pain at 2 weeks (23). For all 3 cases, symptoms resolved after 2–4 months of treatment with no patients experiencing disease recurrence at one year follow up (23). All 3 patients experienced dry lips and mucous membranes, which did not impact their quality of life. Additionally, patients experienced local redness, swelling and pain after PDT which all resolved within 1 week post-treatment.

## Discussion

There is evidence to suggest acupuncture or moxibustion alone or in combination with other treatment interventions may be efficacious for treating numerous etiologies of hair loss. Notably, all studies in this review reported benefits in hair loss and growth in response to treatment. However, the conclusion that can be drawn regarding acupuncture in hair loss are severely limited as the majority of studies did not evaluate acupuncture alone. Only a handful of controlled trials have been performed with the majority of evidence being in case-reports. The topic of acupuncture remains understudied in Western medicine with the majority of studies originating in Eastern countries. Nonetheless, published reports have overwhelmingly showed benefits associated with acupuncture as an adjuvant treatment to both traditional therapies and PDT, warranting further exploration of this treatment modality.

The limited side effect profile of acupuncture makes it an advantageous treatment option for alopecia. As previously mentioned, adverse effects associated with finasteride, a mainstay treatment for both seborrheic and androgenetic alopecia, include erectile dysfunction and decreased arousal secondary to decreased levels of sex hormones (24). Although its existence remains controversial, post-finasteride syndrome, a constellation of sexual, and psychological side effects that persist following cessation of the medication, has also been increasingly reported in the literature (25). As a result, this side effect profile may be prohibitive to its use, particularly in male patients. In contrast, acupuncture is widely viewed as a safe practice when performed by trained practitioners (26). The estimated prevalence of minor adverse events is 1.3 per 1000 treatments, with the most common side effects being nausea, syncope and pain and bruising at the site of application (27). However, a systematic review by Chan et al. showed serious adverse events, although incredibly rare, can include internal or soft tissue injury, infection, and spinal cord injury (28). There is also the potential for underreporting of adverse events in the literature as adverse events are not commonly evaluated in acupuncture studies (28). We found only 2 of the 8 studies assessed the rate of adverse events in their outcomes. Despite the need for better practice in reporting of adverse events, the relative safety of acupuncture has been overwhelmingly supported in the literature and makes it an increasingly appealing treatment option.

### Potential Mechanisms

The potential mechanism of action behind the effect of acupuncture in alopecia may be disease dependent and stem from two different processes that lead to a reduction in inflammation and a decrease in testosterone. Alopecia areata is characterized by an immune-mediated attack on anagen hair follicles and inflammation-induced dystrophy of the hair follicle (29, 30). Like many other diseases in its class, the pathogenesis is theorized to stem from an interaction of genetic, environmental, and immune factors (29, 30). In a study using murine models for alopecia areata, stimulation of the ST 36 with electroacupuncture was associated with a significant reduction of mast cell degranulation around hair follicles and a phenotypical improvement in hair loss when compared to the untreated group (31). Preclinical studies have also shown acupuncture is able to modulate the balance between Th1 and Th2 CD4 + T-cells to obtund the Th1 response and subsequently decrease the release of pro-inflammatory cytokines including tumor necrosis factor (TNF)-alpha (32). Along these lines, a previously published pilot study assessing the role of acupuncture in rheumatoid arthritis, a Th1-predominant autoinflammatory disease, showed acupuncture significantly decreased disease activity with minimal adverse events (33). This relationship between acupuncture and immunomodulation could underlie the benefits associated with its use in alopecia areata.

Acupuncture may also be efficacious for the treatment of androgenetic alopecia through the reduction of testosterone. Androgens modulate the hair growth cycle by binding to receptors at the dermal papillae where testosterone is converted to the more potent dihydrotestosterone (DHT) *via* 5α-reductase (34). DHT is thought to be necessary to the pathogenesis of androgenetic alopecia as areas of hair loss express higher levels of androgen receptors and 5α-reductase. Moreover, individuals that lack 5α-reductase do not experience the hair loss phenotypical of androgenetic alopecia (34). This relationship also underlies the efficacy of finasteride in treating androgenetic hair loss (34). In a study of patients with seborrheic alopecia by Li et al. and a subsequent study of patients with androgenetic alopecia by Gao et al., both authors noted a significant reduction in testosterone in the ACU + TRAD group (14, 22). Similarly, results of a RCT assessing acupuncture vs. traditional Chinese medicine for polycystic ovary syndrome showed both interventions were associated with reduced total testosterone levels (35). This observed association between acupuncture and testosterone could provide a mechanistic explanation for the benefits of acupuncture in androgenetic alopecia if further validated.

### Commonly Targeted Acupoints

Our review found the most commonly targeted acupoints in hair loss were ST 36 (14–17), GV 20 (15, 17, 18), and LR 3 (15–17). Throughout the practice of acupuncture, different acupoints have been implicated in the treatment of a variety of diseases and ailments. Interestingly, ST 36 and LR 3 are amongst the most commonly used acupoints across all diseases (36). GV 20 is associated with relief of headaches, stroke, insomnia, anxiety, and dizziness, through increasing nitric oxide (NO) production and increasing local circulation (37). Given GV 20’s location on the scalp, this enhancement of NO and increased local circulation may explain its positive impact in the treatment of hair loss. Subsequently, ST 36 is a commonly used treat various autoinflammatory conditions including rheumatoid arthritis (38). In addition to significantly reducing mast cell degranulation and alopecia in murine models, ST 36 has also been found to decrease levels of inflammatory cytokines including TNF-alpha, interleukin (IL)-1, and IL-6 (31, 39, 40). *In vitro* studies have demonstrated the role of TNF-alpha, IL-1 alpha, and IL-1 beta in the inhibition of hair growth through their distortion and disruption of the dermal papilla, matrix, and melanocytes. Consequently, these inflammatory cytokines are thought to mediate the pathological changes seen in alopecia areata (41). Lastly, LR 3 is thought to modulate emotion cognition, associative function, and visual function based on functional imaging studies (42). Emotional stress is often reported as a trigger for episodes of alopecia areata and thus, targeting acupoints associated with modulation of these neuronal networks associated with emotional cognition may help decrease hair loss in alopecia areata (43). Nonetheless, more research is needed to determine the specific acupoints most beneficial in treating alopecia and the identifying the etiologies that will have the best response to acupuncture.

### Barriers to Acupuncture

Barriers to the use of acupuncture as a treatment remedy for hair loss are not insignificant and can include treatment costs, time requirements for treatment, limited access to resources and trained providers and patient aversion to needles (44). Cost of acupuncture ranges from $15 to $400 for an initial session and $15 to $300 for each follow up session, with the estimated annual cost spent by patients seeking acupuncture totaling $3.5 billion (45). These costs can be significant when considering the number of sessions ranged from 3 to 50 sessions for the treatment of hair loss. The time required for acupuncture treatment and follow up appointments is also considerably more intensive than the time needed for treatment with oral or topical medications (44). Moreover, the lack of trained physicians, staff, and space for equipment can contribute to difficulty scheduling appointments and accessing acupuncture treatment as well (44). Home treatment may represent a viable alternative to in-office treatment and provide a solution to the cost and time burden but self-administration of acupuncture poses significant training and safety barriers for patients. It should also be noted acupuncture not be an amendable treatment option for all patients, especially those with a phobia of needles (44). However, with the emergence of laser acupuncture, the practice of photonic stimulation of acupoints in absence of needles, serves as a non-invasive alternative to traditional acupuncture (46). Adverse events associated with laser acupuncture were mild and primarily included tingling, transient fatigue, and pain flare-ups which all resolved within 24 h of onset (46). Given the relative safety of laser acupuncture, it may be a safer form of acupuncture for patients in general if shown to be efficacious for hair loss and serve as an alternative for patients with aversion to needles (46).

## Limitations

Only eight clinical studies were identified, therefore the power of this review is limited by the small sample set. Language also served as a major limitation as studies in Chinese and South Korean, were not readily accessed and were not represented in our findings. This barrier is incredibly challenging considering the majority of research in acupuncture originate from Eastern countries. There is one published review focusing on alternative therapies for the treatment alopecia based on studies solely published in South Korean medical journals (47). Similar to our findings, the authors reported the most commonly used acupoints for alopecia included ST 36 and GV 20, which were used in 68 and 23% of eligible studies, respectively (48). Other common acupoints included EX-HN 1 and GB 5 (48). There was also significant heterogeneity in treatment interventions and measurement of outcomes and the absence of standardization of acupuncture techniques and clinical endpoints across the studies included in this review. The majority of studies examined other complementary and alternative medicine practices in combination with acupuncture, making it difficult to determine which treatment elicited improvement in hair loss. However, it should be noted that clinical improvements in hair loss was observed across all articles. Lastly, there were no studies comparing acupuncture with procedural interventions commonly used to treat alopecia including intralesional steroid injections and platelet rich plasma injections and therefore it is unknown how acupuncture compares to these procedural modalities.

## Conclusion

Acupuncture may be an efficacious treatment for numerous etiologies of alopecia. Existing evidence remains limited and heterogenous in protocol and endpoints but overwhelmingly report of benefits associated with acupuncture. This alternative treatment modality continues to be understudied in Western medicine with no existing studies originating in the United States. Yet, given the beneficial side effect profile associated with acupuncture and notable improvements reported in the literature, it is a treatment modality that warrants further exploration. Future directions include performing randomized controlled studies assessing the efficacy of acupuncture alone in comparison to current first-line treatments for alopecia. Additionally, exploring methodologies for safer acupuncture practices, particularly with the application of laser acupuncture, an alternative and non-invasive practice compared to traditional acupuncture, could translate to more accessible treatment and may alleviate the large time and cost barriers needed to undergo acupuncture treatment. Although this acupuncture is viewed as generally safe practice, careful evaluation of adverse events is needed for a better understanding of the side effect profile associated with its practice.

## Author Contributions

AL designed the methodology, performed the data collection, prepared the initial draft, and critically reviewed the manuscript. LA performed the data collection and prepared the initial draft of the manuscript. AH prepared the initial draft of the manuscript. MV formulated the overarching research goals and aims and critically reviewed the manuscript. All authors reviewed the results and approved the final version of the manuscript.

## Conflict of Interest

The authors declare that the research was conducted in the absence of any commercial or financial relationships that could be construed as a potential conflict of interest.

## Publisher’s Note

All claims expressed in this article are solely those of the authors and do not necessarily represent those of their affiliated organizations, or those of the publisher, the editors and the reviewers. Any product that may be evaluated in this article, or claim that may be made by its manufacturer, is not guaranteed or endorsed by the publisher.
